# 

**Published:** 1898-09-10

**Authors:** 


**Notices of Births, Deaths, and Marriages** are inserted in this column at a charge of 2s. 6d. for an announcement not exceeding four lines.

### NOTICE TO CORRESPONDENTS.

**All MS., Letters, Books for Review, and other matters intended for the Editor should be addressed The Editor, "The Hospital," The Hospital Building, 28 & 29, Southampton Street, Strand, W.C.**

The Editor cannot undertake to return rejected MS., even when accompanied by stamped directed envelope.

**Notice to Advertisers and Subscribers.**

All Advertisements, Orders for Copies of The Hospital, and Business Communications should be addressed to **THE MANAGER, (Not to The Editor),** The Hospital, The Hospital Building, 28 & 29, Southampton Street, Strand, London, W.C.

The Cost of Subscription, post free, is as follows:—Per week, 2½d. per quarter, 2s. 8d.; per half-year, 5s. 3d.; per annum, 10s. 6d. Foreign:—Per annum, 15s. 2d.; payable, in all cases, in advance.

**NOTICE.—Vol. XXIII. of "The Hospital" (October, 1897, to March, 1898), in handsome cloth binding, is now on sale at the Office of this Journal, price 7s. 6d. Cases for binding the Numbers contained in this Volume, 1s. 6d. Index to Vol. XXIII., 2½d., post free.**

**The Monthly Part for September is now ready. Price 6d., by post 8d.**

### BOOKS RECEIVED.

BAILLIÈRE, TINDALL, AND COX.

"Cattle Tuberculosis." By T. M. Legge, M.A., M.D., and Harold Sessions, F.R.C.V.S.

HENRY RENSHAW.

"Elements of Pharmacy, Materia Medica, and Therapeutics." By William Whitla, M.A., M.D.

CASSELL AND CO.

"Medical Diseases of Infancy and Childhood." By Dawson Williams, M.D. Lond.

THE SCIENTIFIC PRESS.

"The Bearer's Companion; First Aid to the Injured and Management of the Sick." By E. J. Lawless, M.D.

### The Student of Medicine.

### THE OPENING OF THE MEDICAL SCHOOLS.

The metropolitan medical schools will be opened on the following dates:—

St. George's Hospital on October 1st, with an introductory address at four p.m. by Mr. G. R. Turner. The annual dinner will take place at the Hotel Metropole, at half-past six p.m., when Mr. Timothy Holmes will preside.

The London Hospital Medical College on October 1st. The annual dinner will be held in the college library on October 3rd, when Mr. Mansell-Moullin will take the chair.

Charing Cross Hospital on October 3rd, when Professor Virchow will deliver the second Huxley lecture—"Recent Advances in Science and their Bearing on Medicine and Surgery"—at the St. Martin's Town Hall, Charing Cross. The chair will be taken by Lord Lister at four p.m.

Guy's Hospital on October 3rd. The first meeting of the Physical Society will be held on that day in the new physiological theatre at eight p.m., when Sir Samuel Wilks will preside and a paper will be read by Mr. W. H. Cross. After the meeting a conversazione will be held in the new laboratories and class-rooms. A house dinner of the Students' Club will take place at half-past six p.m. in the college dining-hall.

King's College on October 3rd. The Old Students' dinner will be held at Limmer's Hotel the same evening.

St. Mary's Hospital on October 3rd, with an introductory address at three p.m. by Dr. Caley. The annual dinner will be held in the evening at the King's Hall, Holborn Restaurant, Mr. J. N. Moore in the chair.

Middlesex Hospital on October 3rd. Dr. Voelcker will deliver an introductory address. The annual dinner will take place the same evening at the Café Royal, Regent Street, at seven o'clock, Sir Ralph Thompson in the chair.

St. Thomas's Hospital on October 3rd. The annual dinner will take place the same evening at the Whitehall Rooms, Hôtel Métropole, at six for half-past six, when Dr. Leonard Sedgwick will take the chair.

University College on October 3rd. Introductory lecture by Mr. Sidney Spokes, dental surgeon to the hospital. The annual dinner of the past and present students will take place at the Hotel Cecil at half-past six, Professor F. T. Roberts in the chair.

St. Bartholomew's Hospital on October 3rd. The old students' dinner will be held on October 4th, Sir William Turner in the chair.

Westminster Hospital on October 3rd. The annual dinner will take place in the evening at the Holborn Restaurant, Sir George S. Robertson in the chair.

The London School of Medicine for Women on October 3rd. An introductory address will be delivered by Dr. J. W. Carr, senior assistant physician to the Royal Free Hospital, on October 4th, at eight p.m.

Mason College, Birmingham, on October 1st, when Professor Michael Foster will deliver an address, after which there will be a conversazione.

Yorkshire College, Leeds, on October 3rd, when an address will be delivered and prizes distributed by Dr. C. J. Cullingworth.

The University College of South Wales and Monmouthshire, Cardiff, on October 3rd, and Dr. Robert Saundby will deliver an address on October 7th.

University College, Liverpool, on October 1st. The open-

THE STUDENTS OF MEDICINE—*continued.*

ing ceremony in connexion with the new laboratories of physiology and pathology will take place on October 8th, when Lord Lister will declare the laboratories open.

Owens College, Manchester, on October 3rd.

University of Durham College of Medicine, Newcastle-on-Tyne, on October 1st.

University College, Sheffield, on October 3rd, when Dr. Dyson, vice-president of the college, will deliver the introductory lecture.

### APPOINTMENTS.

F. Barrington, M.B.Edin., F.R.C.S., appointed Honorary Surgeon to the Lewisham Hospital for Women and Children, Sydney. Dr. D. J. Belding, appointed Medical Officer of Health to the Mitford and Launditch Rural District Council. W. E. Collins, M.B.Lond., M.R.C.S.Eng., appointed Honorary Surgeon to the Wellington Hospital, New Zealand. Dr. E. Day, appointed Medical Officer for the Nettlebed Districts of the Henley Union. D. W. Duncan, appointed Medical Officer of Health to the Clay Cross Urban District Council. R. S. C. Edleston, M.R.C.S., L.R.C.P.Lond., appointed Assistant Medical Officer to the Brentford Union Infirmary. W. Edmunds, M.A., M.C., appointed Surgeon to Out-patients on the Honorary Medical Staff of the Evelina Hospital for Sick Children. D. P. James, F.R.C.S.Eng., L.R.C.P.Lond., appointed Honorary Surgeon to the Wellington Hospital, New Zealand. F. P. Kitson, L.R.C.P.Lond., M.R.C.S.Eng., appointed Medical Officer of Health to the Beaminster Rural District Council. C. F. Lethbridge, M.R.C.S.Eng., appointed Officer of Health for the North and West Ridings of Ripon Shire, Victoria. F. W. Mackenzie, M.B., C.M.Edin., appointed Honorary Ophthalmic Surgeon to the Wellington Hospital, New Zealand. G. H. Makins, F.R.C.S., appointed one of the Consulting-Surgeons on the Honorary Medical Staff of the Evelina Hospital for Sick Children. A. Martin, M.D.Lond., M.R.C.S.Eng., appointed Honorary Surgeon to the Wellington Hospital, New Zealand. C. W. Morgan, M.D.Brux., appointed Government Medical Officer and Vaccinator for the District of Eden, New South Wales. W. J. Olivey, M.R.C.S, Eng., appointed Health Officer for Lawlers, Western Australia.

St. Thomas's Hospital.—The following gentlemen have been selected as House Officers from Tuesday, September 6th, 1898:—

House Physicians.—R. W. C. Pierce, M.B., B.Sc.Lond., L.R.C.P., M.R.C.S., L.S.A., D.P.H. Camb. (Extension); J. R. Charles, B.A., M.B., B.C.Camb., L.R.C.P., M.R.C.S.; E. F. Buzzard, B.A., M.B., B.Ch.Oxon.; and H. C. Haslam, M.A., M.B., B.C.Camb., L.R.C.P., M.R.C.S. (2nd Term.)

House Surgeons.—E. H. Cobb, L.R.C.P., M.R.C.S.; A. C. Robinson, L.R.C.P., M.R.C.S.; F. L. A. Greaves, L.R.C.P., M.R.C.S.; and A. G. Greg, B.A.Camb, L.R.C.P., M.R.C.S.

Assistant House Surgeons.—S. O. Bingham, L.R.C.P., M.R.C.S.; E. M. Corner, M.A., M.B., B.C.Camb., B.Sc Lond., L.R.C.P., M.R.C.S.; J. A. Barnes, L.R.C.P., M.R.C.S.; and J. E. Kilvert, L.R.C P., M.R.C.S.

Obstetric House Physicians.—H. F. Shea, M.B., B.S.Durh, L.R.C.P., M.R.C.S., senior; and J. F. McClean, L.R.C.P., M.R.C.S., junior.

Ophthalmic House Surgeons.—S. N. Babington, L.R.C.P., M.R.C.S., senior; and J. S. Hall, L.R.C.P., M.R.C.S, junior.

Clinical Assistants in the Special Department for Diseases of the Throat.—G. B. Thwaites, L.R.C.P., M.R.C.S., and A. D. Cowburn, L.R.C.P., M.R.C.S.

Clinical Assistants in the Special Department for Diseases of the Skin.—S. H. Belfrage, L.R.C.P., M.R.C.S, and A. E. Stevens, M.B.Durh, L.R.C.P., M.R.C.S.

Clinical Assistants in the Special Department for Diseases of the Ear.—F. H. Allfrey, B.A.Camb., L.R.C.P., M.R.C.S. (Extension); and W. J. Galt, B.A.Oxon., L.R.C.P., M.R.C.S.

